# Loss Aversion and Risk Aversion in Non-Clinical Negative Symptoms and Hypomania

**DOI:** 10.3389/fpsyt.2020.574131

**Published:** 2020-09-23

**Authors:** Federica Klaus, Justin R. Chumbley, Erich Seifritz, Stefan Kaiser, Matthias Hartmann-Riemer

**Affiliations:** ^1^ Department of Psychiatry, Psychotherapy, and Psychosomatics, Psychiatric Hospital, University of Zurich, Zurich, Switzerland; ^2^ Jacobs Center for Productive Youth Development, University of Zurich, Zurich, Switzerland; ^3^ Translational Neuromodeling Unit, Institute for Biomedical Engineering, University of Zurich and ETH Zurich, Zurich, Switzerland; ^4^ Division of Adult Psychiatry, Department of Psychiatry, Geneva University Hospitals, Geneva, Switzerland

**Keywords:** loss-aversion, risk-aversion, decision-making, negative symptoms, hypomania

## Abstract

In the field of behavioral decision-making, “loss aversion” is a behavioral phenomenon in which individuals show a higher sensitivity to potential losses than to gains. Conversely, “risk averse” individuals have an enhanced sensitivity/aversion to options with uncertain consequences. Here we examine whether hypomania or negative symptoms predict the degree of these choice biases. We chose to study these two symptom dimensions because they present a common theme across many syndromes with compromised decision-making. In our exploratory study, we employed a non-clinical sample to dissociate the hypomanic from negative symptom dimension regarding choice behavior. We randomly selected a sample of 45 subjects from a student population (18–37 years) without self-reported psychiatric diagnoses (n = 835). We stratified them based on percentiles into a low hypomania/low negative symptoms (n = 15), a hypomania (n = 15), and a negative symptoms group (n = 15) using the hypomanic personality scale (HPS-30) and community assessment of psychic experiences (CAPE). Participants completed a loss aversion task consisting of forced binary choices between a monetary gamble and a riskless choice without gain or loss. We found a reduced loss aversion in participants with higher negative symptoms. In addition, risk aversion was reduced in participants with higher hypomania and negative symptoms compared to low hypomania/negative symptoms. This study adds to the understanding of underlying psychological mechanisms of loss and risk aversion. Given the partially opposing nature of hypomania and negative symptoms, further work is needed to examine whether they affect loss and risk aversion *via* dissociable mechanisms.

## Introduction 

Negative symptoms (apathy, diminished expression) and (hypo-) mania are two, to some extent, opposing symptom dimensions that occur in several psychiatric diseases, such as schizophrenia, depression or bipolar disorder (1). Symptoms from both dimensions can significantly impair everyday functioning (2, 3). Moreover, they have been linked to altered decision-making behavior (4–7), which might partially cause and/or maintain observed symptoms.

Behavioral economics provide powerful methods to investigate variance in decision-making. Recently, these methods have been fruitfully applied in the study of psychopathology (8–10). Negative symptoms and (hypo-) mania have been typically associated with a reduction or an increase, respectively, in the frequency and vigor of goal-directed behavior. Valuation of losses and gains and their accompanying uncertainty constitute core decision-making processes guiding adaptive goal-directed behavior. Thus, these processes might be especially relevant regarding negative and (hypo-) manic symptoms. Clinical observations show an increase in goal-directed behavior and concomitant investment of effort as well as the pursuit of goals without regard to risks in mania (11, 12) and a decrease in goal-directed behavior and invested effort or motivation in negative symptoms (13, 14), both resulting in an impairment of effort-cost computations.

A well-replicated phenomenon in behavioral economics is loss aversion which describes a higher sensitivity to losses than to gains, observable in the general population. In other words, the value of an object is judged higher when it is forgone compared to when it is gained (15, 16). Loss aversion may be understood from an evolutionary point of view (17): when one’s survival is at risk, marginal losses prove more critical for reproductive success than marginal gains (18) and in an environment with low resources, humans are cognitively biased to ensure that they do not fall below some minimal threshold of resources necessary for survival (19). A marked miscalibration of an individual’s loss aversion might impede their social and economic effectiveness and be accompanied by psychopathology.

A reduced or absent sensitivity to loss in schizophrenia patients has been reported, as well as altered risk-taking when decisions are based on prospective outcomes (20–22). Moreover, one study found a significant negative correlation between total symptom severity and loss aversion (21). This contrasts with the idea that an increased loss aversion could diminish goal-directed behavior which in turn manifests itself as negative symptoms. However, no study so far has addressed potential links to specific symptom dimensions, such as negative symptoms. Moreover, to our knowledge no study has investigated loss aversion in (hypo-) manic states, which is surprising considering that the diagnostic criteria for manic episodes (1) include impulsivity and disregard for the potential losses and risks accompanying one’s actions. One study in euthymic bipolar patients using a computerized decision-making task has observed that in positively framed dilemmas, i.e. presented in terms of opportunities to gain rewards, the shift between risk-averse and risk-seeking choices was significantly reduced in bipolar patients. In positively framed dilemmas, the bipolar participants made more risky choices for increased gains while in the negatively framed dilemmas, they made fewer risky choices to avoid losses (23). Another study has found no performance deficit in manic bipolar patients during a probability-based gambling paradigm dependent upon risk-taking situations aimed at maximizing gain and minimizing loss (24). However, the direct link between (hypo-) manic or negative symptom dimensions and the degree of loss and risk aversion remains to be elucidated.

Importantly, features of negative symptoms and (hypo-) mania can be found not only in severely ill patients, but vary along a continuum in the non-clinical population (25, 26). The premise of these dimensional approaches to psychopathology is that clinical and non-clinical symptom expression should at least in part be related to similar underlying mechanisms. Thus, the investigation of the extreme individuals within the “normal” population, being more accessible to study, offers a powerful way to dissect non-clinical and clinical disease mechanisms (27, 28).

The current study investigated whether elevated negative and hypomanic symptoms in a stratified non-clinical sample were associated with differences in decision-making, more specifically loss and risk aversion using a binary choice task with monetary incentives. We did hypothesize that groups with high negative symptoms and/or high hypomania would demonstrate reduced loss and altered risk aversion. As summarized above, a reduced or absent sensitivity to loss and an altered behavioral approach to risk has been reported in schizophrenia of which negative symptoms are one of the main psychopathologic constituents. Regarding (hypo-)mania, its clinical definition includes impulsivity and disregard for potential losses and risks which is a behavior congruent with a reduced aversion to losses.

## Materials and Methods

### Ethics Statement

The studies involving human participants were reviewed and approved by the local ethics committee of the Canton of Zurich. The patients/participants provided their written informed consent to participate in this study. No animal studies are presented in this manuscript. No potentially identifiable human images or data is presented in this study.

### Participants

Eight hundred thirty-five students of the University of Zurich (607 male; age: *M* = 24.3, *SD* = 5.49) were recruited through university mailing lists and social media. After passing an initial screening *via* online questionnaire that asked whether the participants were currently in treatment due to a psychiatric condition and whether they have a psychiatric diagnosis (eligibility criteria: answer “no” to these questions), eligible participants filled out online trait questionnaires. These questionnaires assessed hypomania using the Hypomanic Personality Scale (HPS-30: *M* = 11.1, *SD* = 4.88) (29) and negative symptoms using the negative symptom items of the Community Assessment of Psychic Experiences (CAPE, *M* = 1.62, *SD* = 0.49) (30). As reimbursement they took part in a lottery for Amazon gift cards (300 Swiss Francs, i.e. ~300 USD).

### Procedure

Exclusion criteria were current treatment for a psychiatric disorder (therapy and/or medication), inclusion criteria was being between 18 and 55 years old. On the basis of this large reference population we defined high negative symptoms/low hypomania (“negative symptom group”), high hypomania/low negative symptoms (“hypomania symptom group”), and low negative symptoms/low hypomania (“low hypomania/low negative symptoms group”) target subpopulations. The cut-off for “low” or “high” scores was defined as scores that were below the 40th and above the 60th percentile of the reference population respectively. These cutoffs allowed a clear separation between the three groups while at the same leaving a sufficient pool in each group to conduct random sampling of subjects invited to participate in the experimental tasks.

In clinical populations, the co-occurrence of hypomania/mania and negative symptoms is very rare, we therefore decided not to include such a group.

In a second step, participants meeting the target criteria were contacted randomly based on a random number generator [MATLAB *rand* function, 2012 (31)] *via* e-mail and invited to the laboratory: The three equal groups were derived by contacting 15 participants from each strata, uniformly at random. Upon reaching of 15 enrolled participants per group, the recruitment process was stopped and this resulted in n = 45 participants in total. Please note that based on the exploratory nature of the study and the limited literature, we refrained from conducting a power analysis (32).

The reference sample (n = 835), the stratification, and the recruited study participants are depicted in **Supplementary Figure S1**.

### Experimental Task

Participants were endowed with 30 CHF at the beginning of the study. Participants then completed a loss aversion task (33), consisting of 20 forced binary choices between a monetary gamble (P = 0.5) and a riskless choice with no gain/loss (P = 1). Participants were presented on a computer screen three numbers representing amounts of money, two of them representing the possible gain or loss in case the gamble was accepted and one representing 0 CHF in case of a rejection of the gamble (**Supplementary Figure S2**). Following standard practice in behavioral economics, subjects knew in advance that their final payment would equal their 30 CHF endowment, plus or minus one of their 20 outcomes, selected at random. The outcome of each chosen gamble was revealed to the subject immediately. At the end of the experiment, they were then paid according to the policy just detailed above, debriefed, and dismissed. We used their choices to quantify a subject-specific loss parameter, called λ below. In addition, choices were used to quantify attitudes toward chance (risk aversion ρ) and consistency over choices (logit sensitivity μ).

### Data Analysis

We used a three-parameter model to estimate choice behavior: Gains were estimated by exploiting equation 1: $u(x^{+})x^{\rho }$, and losses via equation 2: $u(x^{-})=-\lambda *{\left(-x\right)}^{\rho }$. These combine in equation 3: $p(gambleacceptance)={\left(1+exp\{-\mu (u(gamble)-u(guaranteed))\}\right)}^{-1}$, where *u*(*gamble*) is the difference between equations 1 and 2. *u* indicates the anticipated utility or “desirability,” *x* indicates the (monetary) value; *ρ* is the curvature of the utility function and indicates the risk aversion, *λ* is the loss aversion coefficient and refers to the multiplicative valuation of losses relative to gains (with *λ*>1: loss aversion, *λ*<1: gain seeking, *λ*=0: gain-loss neutral), *p* indicates the probability of an event, and *μ* indicates the sensitivity of the participant’s choices to changes in the difference between subjective values of the gamble and the guaranteed fixed amount (consistency). The subject-specific parameter *λ*, our main outcome representing loss aversion, was estimated from subject-specific expected marginal posteriors. Parameters *μ* and *ρ* were estimated in the same way. For details see (33–35). To determine the between-subject differences, a one-way multivariate analysis of variance using IBM SPSS 25 was performed with *λ, μ* and *ρ* as dependent measures and group as independent measure. Age was introduced as covariate, due to the exploratory nature of this study *post-hoc* testing was performed using LSD (least significant difference), Cohen’s d and partial eta square (η^2^) was used to estimate effect sizes.

## Results

### Sample Characteristics

The three groups did not differ significantly in gender (*F*(2,42) = .368, *p* = .694), income (*F*(2,42) = .883, *p* = .421), education (*F*(2,42) = 1.451, *p* = .246), or money spent per month (*F*(2,42) = 1.037, *p* = .363). However, a significant group difference regarding age was observed (*F*(2,42) = 4.055, *p* = .025). The hypomania group (age: *M* = 21.80, *SD* = 2.24, age range: 18–27 years) was significantly younger than the negative symptom (age: *M* = 25.93, *SD* = 5.26, age range: 20–37 years) group with a mean difference of 4.13 years (*p* = .007), while the negative symptom and the hypomania group did not differ in age in comparison to the low hypomania/negative symptom group (age: *M* = 23.80, *SD* = 3.84, age range: 20–31 years) (*p* = .149 and *p* =. 176 respectively).

Based on the observed group difference in age, we included age as covariate in our analyses below.

### Loss Aversion Is Reduced in Negative Symptoms

There was a highly significant main effect of group on loss aversion (see **Table 1**). *Post-hoc* testing revealed a significant difference between the negative symptom group and low hypomania/negative symptom group with lower loss aversion in the negative symptom group. Further, a significant difference between the hypomania group and negative symptom group was present, with higher loss aversion in the hypomania group compared to the negative symptom group. There was a trend-level difference with reduced loss aversion in the hypomania group compared to the low hypomania/negative symptom group. A trend-level significance level was defined as p >0.05 and <0.06.

**Table 1:** Sample Characteristics of Participant Groups, Means, Standard Deviations, and One-Way Analyses of Covariance in Decision-Making Experiment.

Measure	Low hypomania/negative symptom group		Negative symptom group		Hypomania group					
	*n*	*%*	*n*	*%*	*n*	*%*				
Gender										
Female	6	40	4	27	6	40				
Male	9	60	11	73	9	60				
										
	*M*	*SD*	*M*	*SD*	*M*	*SD*				
CAPE score	1.11	0.15	2.23	0.43	1.15	0.17				
HPS-30 score	5.73	1.71	5.87	1.64	16.93	2.49				
										
	**Low hypomania/negative symptom group (1)**		**Negative symptom group (2)**		**Hypomania group** **(3)**		***F*(2, 41)**	**η^2^**	***Post-hoc***	**Cohen’s d**
	***M***	***SD***	***M***	***SD***	***M***	***SD***				
Loss aversion	2.56	0.84	1.53	0.59	1.96	0.69	9.51***	0.32	2<3* 2<1***	0.86 1.60
Risk aversion	0.88	1.77	1.18	0.28	1.08	0.3	4.76*	0.19	2<1** 3<1*	1.02 0.88
Consistency	2.05	0.61	1.79	0.88	1.58	0.82	1.86	0.83		

*p < .05. **p < .01. ***p < .001.

N = 45 (n = 15 for each group). Age introduced as covariate in analysis. Only significant results of post-hoc comparison are reported. The numbers in parentheses in group names refer to the numbers used in illustrating statistically significant differences. Effect sizes (η^2^, Cohen’s d) can be interpreted as small (0.2), medium (0.5), and large (0.8) (36).

CAPE, Community Assessment of Psychic Experiences; HPS-30, Hypomanic Personality Scale.

### Risk Aversion Is Reduced in Negative Symptoms and Hypomania

We observed a significant main effect of group on risk aversion. *Post-hoc* testing revealed less risk aversion in participants with higher negative symptoms or hypomania compared to low hypomania/negative symptom and no difference between symptom groups (see **Table 1** for details).

### Consistency Does Not Differ Between Negative Symptoms, Hypomania, and Low Symptoms

No significant difference was observed regarding consistency (no significant main effect of group) (see **Table 1** for details).

## Discussion

Our work is the first to investigate loss and risk aversion in a non-clinical population with the two symptom dimensions of negative symptoms and hypomania. We reported evidence of a significant reduction in loss aversion in participants with non-clinical negative symptoms, and a trend-level reduction in participants with hypomania symptoms after adjustment for age. We further report that risk aversion is similarly diminished in participants with non-clinical negative symptoms and hypomania symptoms. This suggests that these distinct symptom dimensions manifest in a common behavioral phenotype. We will now discuss our findings for negative and hypomania symptoms, before attempting a synthesis of our pattern of results.

The finding that the *high negative symptom* group shows a reduced loss and risk aversion in our study may be important for understanding previous findings on loss and risk aversion in patients with schizophrenia (where negative symptoms are central). Diminished *loss aversion* in patients with schizophrenia in comparison to healthy control participants has been demonstrated previously (21, 22). The reduction in loss aversion was found to correlate with age, duration of illness and hospitalization, and poorer cognitive performance, but not with current psychopathology in one study (22). Conversely, Trémeau et al. found a negative correlation between the extent of loss aversion with schizophrenia severity such that less ill patients showed a loss aversion more similar to controls. This correlation with loss aversion was present for positive symptoms but not negative symptoms (21). Brown et al. found a reduced sensitivity to losses (increased tendency to gamble when faced with a certain loss) in patients with schizophrenia compared to healthy controls (20). Nevertheless, the same study also demonstrated an even greater reduction in sensitivity to gains, which casts doubt on the observed differences in loss aversion between schizophrenia patients and controls (21). One explanation for a reduced loss aversion in negative symptoms in schizophrenia is offered by the theory of altered salience processing, i.e. abnormal weighing of stimuli (21, 37). In other words, the salience of loss is reduced *via* competitive interference from other external and internal stimuli. This might impede a motivational behavior directed at avoiding losses. This hypothesis is supported by the lack of a framing effect in schizophrenia patients (20), i.e. the context seems not to influence the decision-making in schizophrenia as much as it does in healthy controls. Additionally, patients suffering from primary negative symptoms exhibit a reduced sensitivity to negative (distressing) stimuli (38), which might point to underlying mechanisms at play of reactivity to loss.

Similar to loss aversion, the concept of *risk aversion* can be interpreted in the context of an aberrant salience hypothesis for negative symptoms, where the detection of risk is also impaired due to noise, similar to the detection of potential loss. In contrast to our findings, an increased risk aversion was demonstrated in schizophrenia compared to healthy control participants (39) as well as an absence of modulation of *risk-seeking* behavior depending on the framing of the situation, i.e. participants with schizophrenia demonstrated a similar risk-seeking behavior in loss and keep frames (20). Possible explanations for these observations can be the influence of the context or “frame” in which a decision is taken (40).

To summarize, reduced sensitivity to losses was demonstrated by some studies to be a feature of schizophrenia in line with our findings of reduced loss aversion in a stratified non-clinical negative symptom group. However, the relationship with specific symptom domains, specifically negative symptoms, remains to be further investigated. Regarding risk aversion, increased risk aversion and reduced situation-dependency have been observed previously in schizophrenia, a discrepancy to our findings possibly due to the difference in clinical *vs.* non-clinical populations. Further, it has to be considered that different task paradigms and scales to assess symptoms were used, which might complicate the interpretation and comparability of results and also explain the discrepant findings of loss and risk (20).

The absence of a significant reduction of loss aversion in the *hypomania* group compared to the low negative symptom/hypomania group is surprising, given the diagnostic definition of a manic episode, which includes impulsivity and therefore suggests reduced loss and risk aversion (1). Our finding might be due to the small sample size in our study. The hypothesis of a reduced loss aversion is supported by the finding that reduced attention to losses relative to gains (as in mania) is necessary for reduced loss aversion (41). Interestingly, manic patients show a reduced perception of loss as such (42, 43), a feature that is comparable to the absence of distress in schizophrenia (38). This supports our findings of a significant reduction of loss aversion in negative symptoms and a trend-level reduction in mania. Nevertheless, the relationship between manic episodes and loss aversion needs further investigation, including also repetitive assessments in different psychopathologic states over time. Our finding of reduced *risk aversion* in the hypomania group is however fully in line with our hypotheses based on the literature and clinical diagnostic criteria of mania (1, 42).

Our findings of similar directions of altered loss and risk aversion in non-clinical *negative symptoms and (hypo-)mania* seem counter-intuitive at first glance, based on the opposing nature in terms of activity and hedonia level between negative symptoms and (hypo-)mania (1). However, the previously cited literature is in support of our findings. Behavioral finance theories investigating portfolio selection strategies suggest that depressed patients aim for a minimal loss, while manic patients aim for a maximal gain, indifferent of incurring losses in their choice behavior (43, 44). There is a high clinical overlap and challenges in diagnostic differentiation between negative symptoms and depression. Therefore, based on the above outlined theory, once could have expected an increased loss and risk aversion in negative symptoms and a reduced loss and risk aversion in mania, of which we only demonstrate the latter. The finding of a reduced loss aversion in both groups might be accompanied by separate underlying mechanisms. It may also be relevant that the above mentioned studies considered patients, while the present study investigated a non-clinical student sample.

Our study has some *limitations*. The sample size was small so our results require replication. Another limitation pertains to the selection of our study population, which consists of fairly young students. Therefore, our findings cannot be generalized to the general population or to patients with psychiatric diagnoses. Furthermore, we did not collect data on ethnicity and non-clinical positive symptoms. Additionally, the method of selection for the experimental group is limited in its percentile-based approach, which does not account for other variables and bears the risk of selection bias. Further, we did not conduct a standardized psychiatric screening interview, but used self-declaration of the participants regarding being in psychiatric treatment or having a psychiatric diagnosis.

Despite these limitations, we believe that the current study presents an important contribution to the investigation of altered decision-making in non-clinical populations. A dimensional approach to studying psychological concepts, e.g. by investigating populations without a psychiatric diagnosis but with some accentuation in symptom domains, will allow us to understand the mechanisms that contribute to behavioral symptoms that in a non-severe form do not constitute a pathology, but in their severe manifestation can be part of a psychiatric condition (45). We hereby hope to contribute to a basis for future investigation of common and divergent mechanisms in decision-making with relevance to the understanding and potential treatment of altered decision-making in psychiatric conditions.

We demonstrated a reduced loss and risk aversion in the negative symptom and reduced risk aversion in the hypomania dimension. This suggests that these distinct symptom dimensions might manifest a common behavioral phenotype which is highly relevant to the understanding of altered decision-making. It would be of high interest in a future study to investigate whether a similar pattern of loss and risk aversion can be observed in clinical (hypo-)mania or schizophrenia with negative symptoms.

## Data Availability Statement

The datasets presented in this study can be found in online repositories. The names of the repository/repositories and accession number(s) can be found below: https://doi.org/10.7910/DVN/W2KN1M.

## Ethics Statement

The studies involving human participants were reviewed and approved by Local ethics committee of the Canton of Zurich, Switzerland. The patients/participants provided their written informed consent to participate in this study.

## Author Contributions

Data was collected by MH-R. The study was designed by MH-R and SK. Data analyses were performed by FK. All authors assisted in data interpretation. FK drafted the manuscript, and JC, ES, MH-R, and SK provided critical revisions. All authors contributed to the article and approved the submitted version.

## Funding

Volkswagen foundation (Hannover, Germany) through the European Platform for Life Sciences, Mind Sciences and the Humanities (Grant-Nr.: 85061) to SK. Swiss National Science Foundation to SK (Grant-Nr: PP00P1_128574) and to FK (Gran-Nr: P2ZHP3_181506). The funders had no role in study design, data collection and analysis, decision to publish, or preparation of the manuscript.

## Conflict of Interest

The authors declare that the research was conducted in the absence of any commercial or financial relationships that could be construed as a potential conflict of interest.

## Supplementary Material

Supplementary Figure S1 Sample stratification procedure. On the basis of a large reference population (*N* = 835) we defined high negative symptoms/low hypomania (“negative symptom group”), high hypomania/low negative symptoms (“hypomania symptom group”), and low negative symptoms/hypomania target subpopulations. The cut-off for “low” or “high” scores was defined as scores that were below the 40th and above the 60th percentile of the reference population respectively (grey lines).

Supplementary Figure S2 Gambling task. Typical computer screen: numbers represent money in CHF. Subjects had to choose on each trial, between no payoff (reject) and a gamble in which they had an equal chance of winning or losing the specified amounts (accept).
