# Supplementary figures and images for: Loss Aversion and Risk Aversion in Non-Clinical Negative Symptoms and Hypomania
Source: Front Psychiatry. 2020 Sep 23;11:574131. doi: 10.3389/fpsyt.2020.574131 (PMC7538829; doi:10.3389/fpsyt.2020.574131)

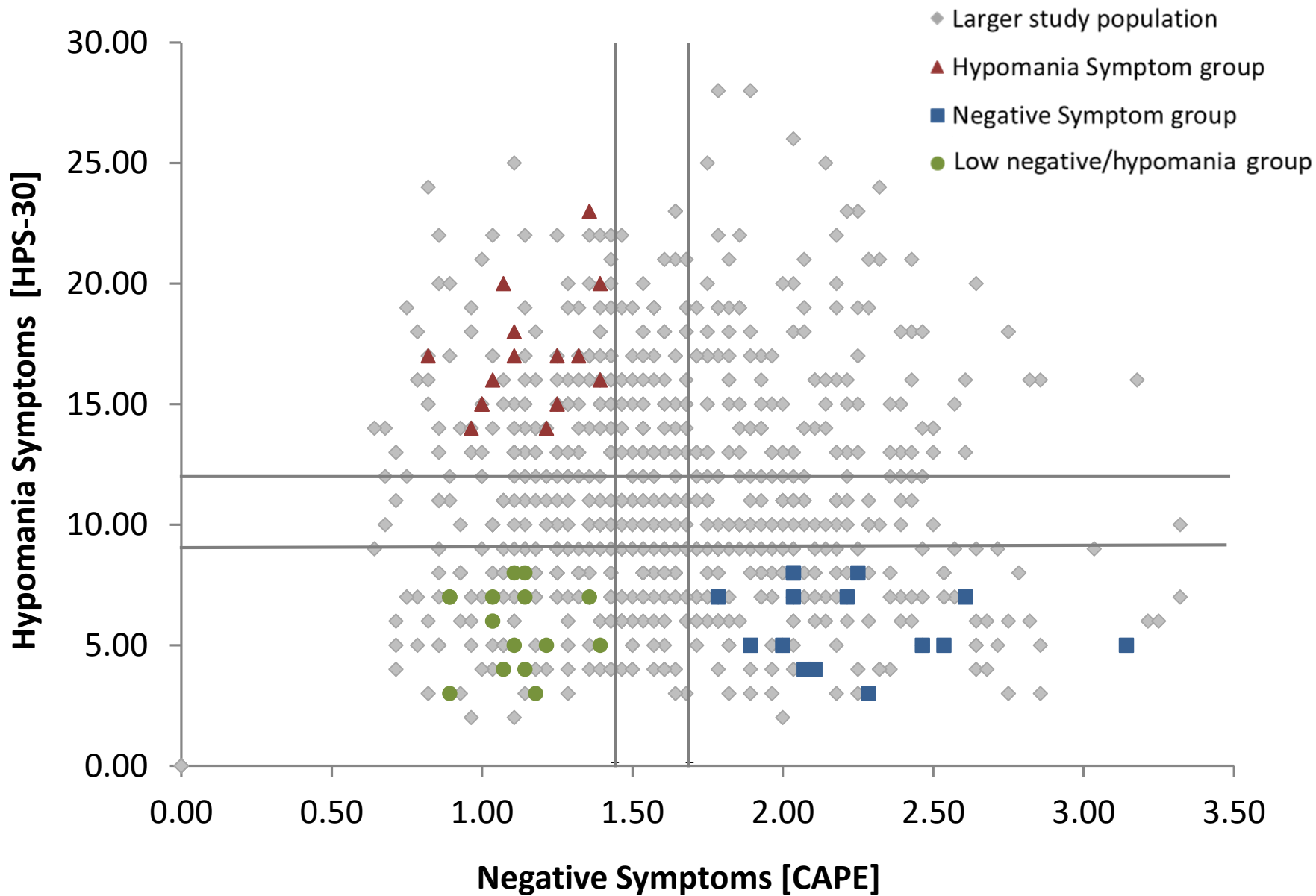

Supplement: Supplementary Figure S1 — Sample stratification procedure. On the basis of a large reference population (N = 835) we defined high negative symptoms/low hypomania (“negative symptom group”), high hypomania/low negative symptoms (“hypomania symptom group”), and low negative symptoms/hypomania target subpopulations. The cut-off for “low” or “high” scores was defined as scores that were below the 40th and above the 60th percentile of the reference population respectively (grey lines). [file DataSheet_1.pdf]
