# Supplementary material for: Loss Aversion and Risk Aversion in Non-Clinical Negative Symptoms and Hypomania
Source: Front Psychiatry. 2020 Sep 23;11:574131. doi: 10.3389/fpsyt.2020.574131 (PMC7538829; doi:10.3389/fpsyt.2020.574131)
Supplement: Supplementary Figure S2 — Gambling task. Typical computer screen: numbers represent money in CHF. Subjects had to choose on each trial, between no payoff (reject) and a gamble in which they had an equal chance of winning or losing the specified amounts (accept). [file DataSheet_2.pdf]

+ 2 CHF

- 1 CHF

or

0 CHF

accept

reject
